# Supplementary material for: Comparative Efficacy and Safety of JAK Inhibitors in the Management of Rheumatoid Arthritis: A Network Meta-Analysis
Source: Pharmaceuticals (Basel). 2025 Jan 28;18(2):178. doi: 10.3390/ph18020178 (PMC11859343; doi:10.3390/ph18020178)
Supplement: Supplementary file 1 [file pharmaceuticals-18-00178-s001.zip › pharmaceuticals-3289758-supplementary.pdf]

Supplementary Table 1: Netleague table describing infection risk and herpes zoster risk by JAKinib treatment.

	Infection risk						
Herpes zoster	**Baricitinib**	2.05 (0.41 - 10.25)	1.32 (0.76 - 2.27)	0.98 (0.72 - 1.35)	1.11 (0.82 - 1.51)	1.19 (0.91 - 1.55)	1.29 (1.11 - 1.49)
	-	**Decernotinib**	0.64 (0.12 - 3.47)	0.48 (0.09 - 2.45)	0.54 (0.11 - 2.76)	0.58 (0.11 - 2.93)	0.63 (0.13 - 3.13)
	1.29 (0.05 - 35.26)	-	**Filgotinib**	0.75 (0.41 - 1.36)	0.85 (0.47 - 1.52)	0.90 (0.51 - 1.60)	0.98 (0.58 - 1.65)
	8.04 (0.97 - 66.53)	-	6.24 (0.23 - 171.96)	**Peficitinib**	1.13 (0.77 - 1.66)	1.21 (0.84 - 1.73)	1.31 (0.99 - 1.73)
	4.41 (0.59 - 33.27)	-	3.42 (0.13 - 89.01)	1.11 (0.19 - 6.41)	**Tofacitinib**	1.07 (0.75 - 1.51)	1.16 (0.89 - 1.51)
	4.91 (0.78 - 30.95)	-	3.81 (0.16 - 88.94)	0.61 (0.10 - 3.91)	1.11 (0.19 - 6.41)	**Upadacitinib**	1.08 (0.87 - 1.35)
	4.46 (1.01 - 19.69)	-	3.46 (0.18 - 66.53)	0.55 (0.12 - 2.50)	1.01 (0.26 - 3.98)	0.91 (0.31 - 2.69)	**Placebo**

Supplementary Table 2: Netleague table describing infection risk and herpes zoster risk by JAKinib treatment.

	Thrombotic risk						
Malignancy risk	**Baricitinib**	0.51 (0.02 - 13.74)	1.32 (0.12 - 14.05)	-	2.83 (0.26 - 31.14)	0.49 (0.05 - 5.06)	1.15 (0.25 - 5.31)
	-	**Decernotinib**	2.58 (0.08 - 78.95)	-	5.53 (0.18 - 173.25)	0.96 (0.03 - 28.71)	2.24 (0.12 - 41.10)
	-	-	**Filgotinib**	-	2.15 (0.16 - 28.21)	0.37 (0.03 - 4.61)	0.87 (0.14 - 5.26)
	-	-	-	**Peficitinib**	-	-	-
	1.72 (0.18 - 16.81)	-	-	-	**Tofacitinib**	0.17 (0.01 - 2.21)	0.40 (0.06 - 2.56)
	1.70 (0.21 - 14.10)	-	-	-	0.99 (0.09 - 11.12)	**Upadacitinib**	2.34 (0.40 - 13.64)
	1.13 (0.28 - 4.49)	-	-	-	0.66 (0.11 - 4.02)	0.66 (0.13 - 3.28)	**Placebo**

Supplementary table 3: Netleague table describing MACE risk and hypercholesterolemia risk by JAKinib treatment.

	MACE risk						
Hypercholesterolemia	**Baricitinib**	0.96 (0.03 - 28.18)	2.44 (0.25 - 23.73)	2.26 (0.14 - 35.50)	1.79 (0.18 - 17.81)	2.07 (0.29 - 14.62)	0.97 (0.24 - 3.86)
	0.48 (0.06 - 3.90)	**Decernotinib**	2.53 (0.07 - 89.98)	2.35 (0.05 - 115.20)	1.86 (0.05 - 66.97)	2.15 (0.07 - 62.88)	1.01 (0.05 - 21.89)
	0.36 (0.02 - 6.39)	0.75 (0.02 - 23.78)	**Filgotinib**	0.93 (0.05 - 18.45)	0.73 (0.06 - 9.65)	0.85 (0.09 - 8.27)	0.40 (0.07 - 2.43)
	-	-	-	**Peficitinib**	0.79 (0.04 - 15.99)	0.92 (0.06 - 14.37)	0.43 (0.04 - 4.64)
	0.59 (0.13 - 2.80)	1.24 (0.11 - 14.54)	1.64 (0.07 - 38.47)	-	**Tofacitinib**	1.16 (0.12 - 11.53)	0.54 (0.09 - 3.40)
	0.72 (0.03 - 15.76)	1.50 (0.04 - 56.70)	1.99 (0.03 - 123.98)	-	1.21 (0.04 - 34.51)	**Upadacitinib**	0.47 (0.12 - 1.86)
	1.78 (0.97 - 3.27)	3.71 (0.50 - 27.63)	4.93 (0.30 - 81.94)	-	3.00 (0.72 - 12.54)	2.48 (0.12 - 51.39)	**Placebo**

Supplementary Table 4: PRISMA checklist 2020.

Section and Topic	Item #	Checklist item	Location where item is reported
**TITLE**			
Title	1	Identify the report as a systematic review.	1
**ABSTRACT**			
Abstract	2	See the PRISMA 2020 for Abstracts checklist.	1
**INTRODUCTION**			
Rationale	3	Describe the rationale for the review in the context of existing knowledge.	2
Objectives	4	Provide an explicit statement of the objective(s) or question(s) the review addresses.	2
**METHODS**			
Eligibility criteria	5	Specify the inclusion and exclusion criteria for the review and how studies were grouped for the syntheses.	14
Information sources	6	Specify all databases, registers, websites, organisations, reference lists and other sources searched or consulted to identify studies. Specify the date when each source was last searched or consulted.	14
Search strategy	7	Present the full search strategies for all databases, registers and websites, including any filters and limits used.	14
Selection process	8	Specify the methods used to decide whether a study met the inclusion criteria of the review, including how many reviewers screened each record and each report retrieved, whether they worked independently, and if applicable, details of automation tools used in the process.	14
Data collection process	9	Specify the methods used to collect data from reports, including how many reviewers collected data from each report, whether they worked independently, any processes for obtaining or confirming data from study investigators, and if applicable, details of automation tools used in the process.	14
Data items	10a	List and define all outcomes for which data were sought. Specify whether all results that were compatible with each outcome domain in each study were sought (e.g. for all measures, time points, analyses), and if not, the methods used to decide which results to collect.	14
	10b	List and define all other variables for which data were sought (e.g. participant and intervention characteristics, funding sources). Describe any assumptions made about any missing or unclear information.	14
Study risk of bias assessment	11	Specify the methods used to assess risk of bias in the included studies, including details of the tool(s) used, how many reviewers assessed each study and whether they worked independently, and if applicable, details of automation tools used in the process.	15
Effect measures	12	Specify for each outcome the effect measure(s) (e.g. risk ratio, mean difference) used in the synthesis or presentation of results.	15
Synthesis methods	13a	Describe the processes used to decide which studies were eligible for each synthesis (e.g. tabulating the study intervention characteristics and comparing against the planned groups for each synthesis (item #5)).	15

Section and Topic	Item #	Checklist item	Location where item is reported
	13b	Describe any methods required to prepare the data for presentation or synthesis, such as handling of missing summary statistics, or data conversions.	15
	13c	Describe any methods used to tabulate or visually display results of individual studies and syntheses.	15
	13d	Describe any methods used to synthesize results and provide a rationale for the choice(s). If meta-analysis was performed, describe the model(s), method(s) to identify the presence and extent of statistical heterogeneity, and software package(s) used.	15
	13e	Describe any methods used to explore possible causes of heterogeneity among study results (e.g. subgroup analysis, meta-regression).	15
	13f	Describe any sensitivity analyses conducted to assess robustness of the synthesized results.	NR
Reporting bias assessment	14	Describe any methods used to assess risk of bias due to missing results in a synthesis (arising from reporting biases).	15
Certainty assessment	15	Describe any methods used to assess certainty (or confidence) in the body of evidence for an outcome.	15
**RESULTS**			
Study selection	16a	Describe the results of the search and selection process, from the number of records identified in the search to the number of studies included in the review, ideally using a flow diagram.	3
	16b	Cite studies that might appear to meet the inclusion criteria, but which were excluded, and explain why they were excluded.	3
Study characteristics	17	Cite each included study and present its characteristics.	3
Risk of bias in studies	18	Present assessments of risk of bias for each included study.	7
Results of individual studies	19	For all outcomes, present, for each study: (a) summary statistics for each group (where appropriate) and (b) an effect estimate and its precision (e.g. confidence/credible interval), ideally using structured tables or plots.	2-12
Results of syntheses	20a	For each synthesis, briefly summarise the characteristics and risk of bias among contributing studies.	7
	20b	Present results of all statistical syntheses conducted. If meta-analysis was done, present for each the summary estimate and its precision (e.g. confidence/credible interval) and measures of statistical heterogeneity. If comparing groups, describe the direction of the effect.	2-12
	20c	Present results of all investigations of possible causes of heterogeneity among study results.	2-12
	20d	Present results of all sensitivity analyses conducted to assess the robustness of the synthesized results.	NR
Reporting biases	21	Present assessments of risk of bias due to missing results (arising from reporting biases) for each synthesis assessed.	7

Section and Topic	Item #	Checklist item	Location where item is reported
Certainty of evidence	22	Present assessments of certainty (or confidence) in the body of evidence for each outcome assessed.	2-12
**DISCUSSION**			
Discussion	23a	Provide a general interpretation of the results in the context of other evidence.	13-14
	23b	Discuss any limitations of the evidence included in the review.	13-14
	23c	Discuss any limitations of the review processes used.	13-14
	23d	Discuss implications of the results for practice, policy, and future research.	13-14
**OTHER INFORMATION**			
Registration and protocol	24a	Provide registration information for the review, including register name and registration number, or state that the review was not registered.	NR
	24b	Indicate where the review protocol can be accessed, or state that a protocol was not prepared.	NR
	24c	Describe and explain any amendments to information provided at registration or in the protocol.	NR
Support	25	Describe sources of financial or non-financial support for the review, and the role of the funders or sponsors in the review.	16
Competing interests	26	Declare any competing interests of review authors.	16
Availability of data, code and other materials	27	Report which of the following are publicly available and where they can be found: template data collection forms; data extracted from included studies; data used for all analyses; analytic code; any other materials used in the review.	16

Supplementary Table 5: Search strategies in different databases

Search No	PubMed Search Query	Results
1	(tofacitinib or cp 690 550 or cp 690550 or cp690 550 or cp690550 or tasocitinib or tofacitinib or xeljanz)	3,138
2	(baricitinib or incb 28050 or ly 3009104 or ly3009104 or olumiant)	1,492
3	(upadacitinib or ABT-494 or Rinvoq)	748
4	(decernotinib or VX-509)	34
5	(peficitinib or ASP015K or Smyraf)	114
6	(filgotinib or GS-6034 or GLPG0634 or GLPG0634)	313
7	#1 OR #2 OR #3 OR #4 OR #5 OR #6	4,889
8	arthritis, rheumatoid[MeSH Terms]	1,27,224
9	"Rheumatoid arthritis"	1,53,758
10	#8 or #9	1,69,057
11	#7 and #10	1,559
12	#7 and #10 (filter applied: Clinical trials)	**219**
**CENTRAL Search Query**		
1	(tofacitinib or cp 690 550 or cp 690550 or cp690 550 or cp690550 or tasocitinib or tofacitinib or xeljanz):ti,ab,kw	1,189
2	(baricitinib or incb 028050or incb 28050 or incb028050 or incb28050 or ly 3009104 or ly3009104 or olumiant):ti,ab,kw	706
3	(upadcitinib or ABT-494 or Rinvoq):ti,ab,kw	104
4	(decernotinib or VX-509):ti,ab,kw	26
5	(peficitinib or ASP015K or Smyraf):ti,ab,kw	55
6	(filgotinib or GS-6034 or GLPG0634 or GLPG0634):ti,ab,kw	370
7	#1 or #2 or #3 or #4 or #5 or #6	2,408
8	("rheumatoid arthritis"):ti,ab,kw	18,419
9	#7 and #8	**1,169**

Supplementary Figures

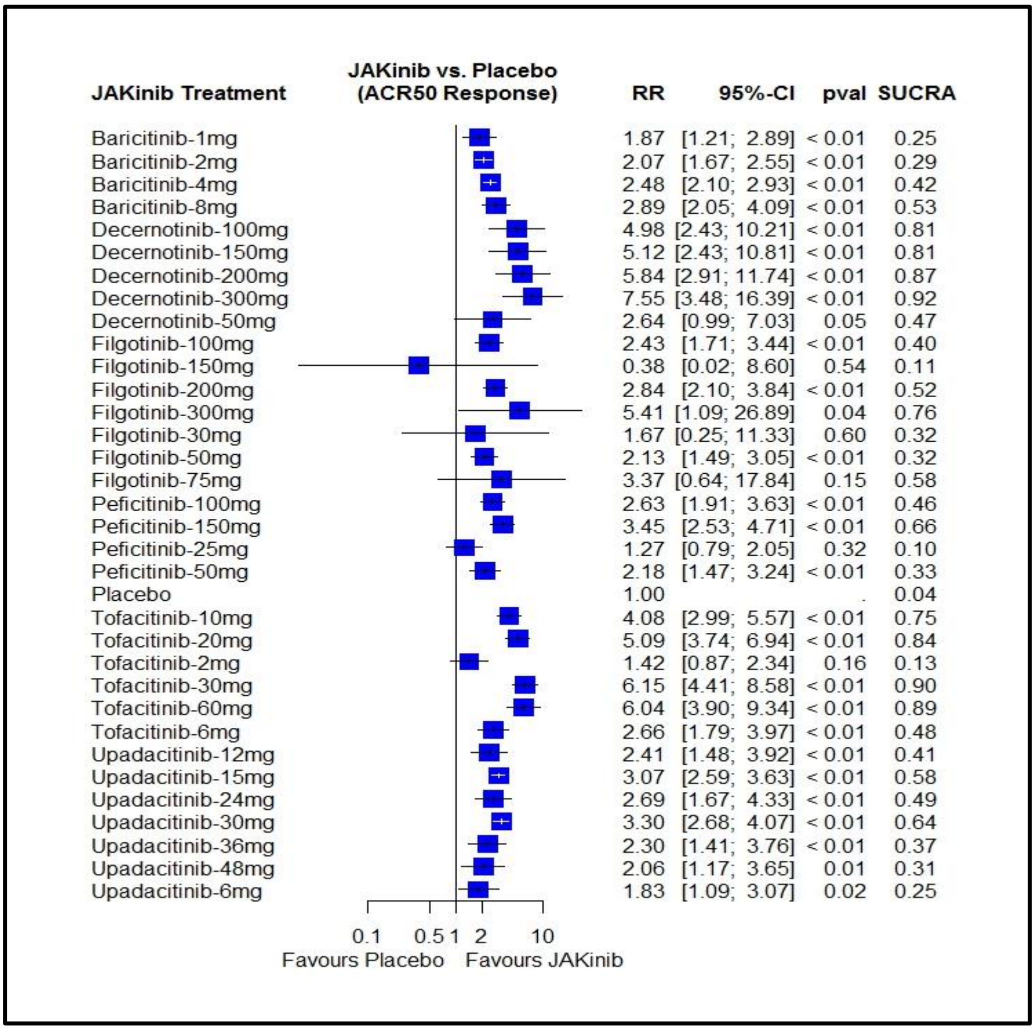

Supplementary Figure 1: Forest plot for ACR50 response by different doses (mg) of JAKinibs treatment.

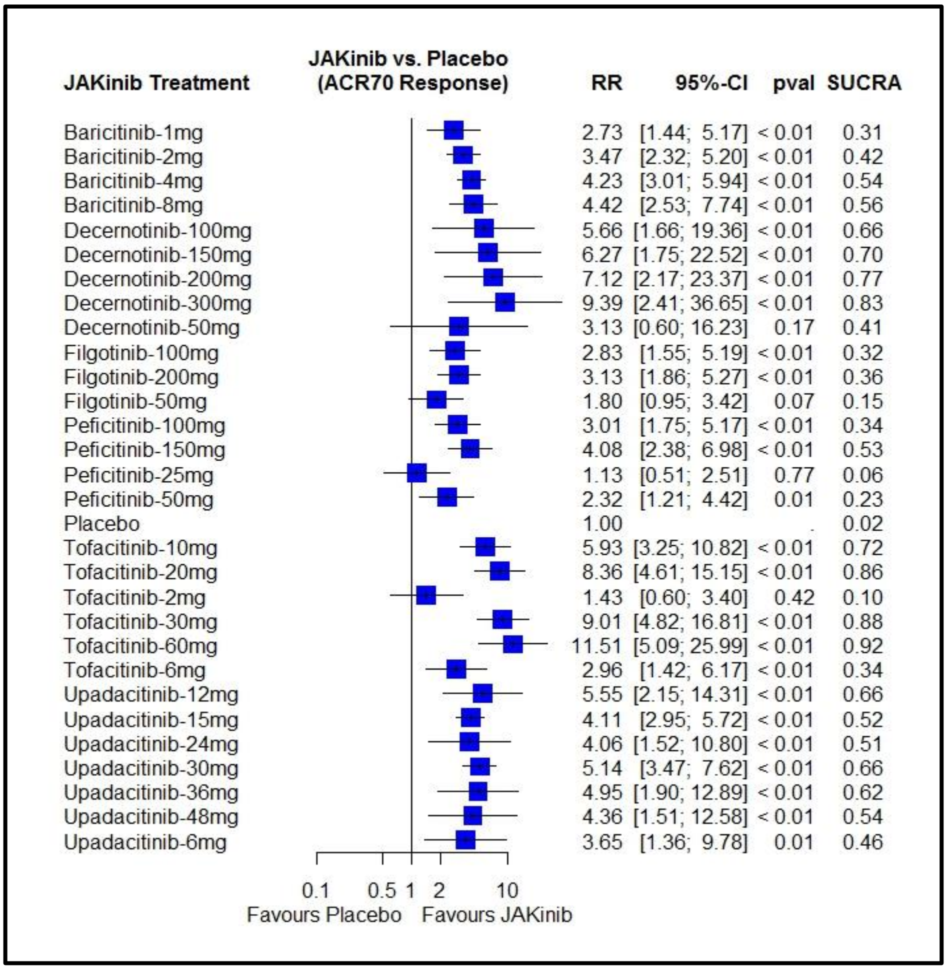

Supplementary Figure 2: Forest plot for ACR70 response by different doses (mg) of JAKinibs treatment.

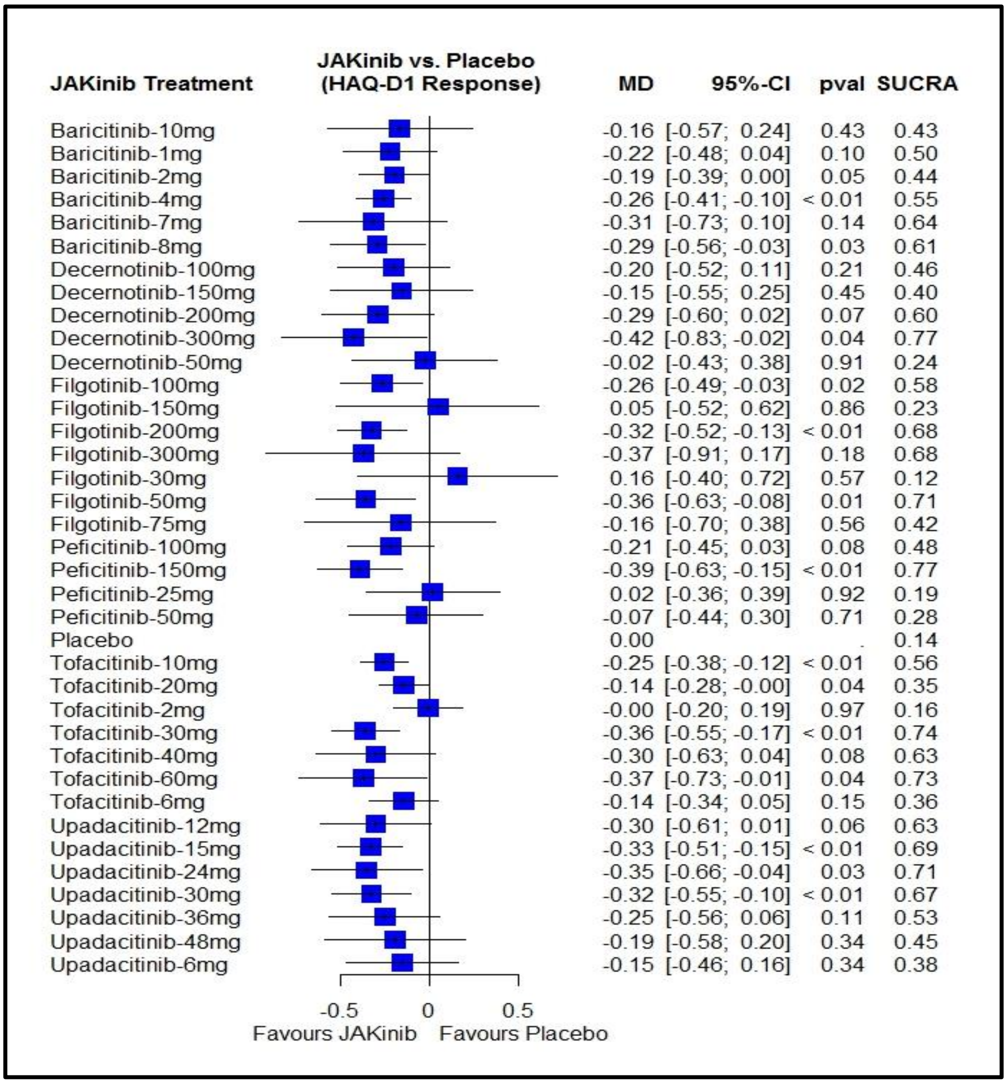

Supplementary Figure 3: Forest plot for HAQ-D1 response by different doses (mg) of JAKinibs treatment.

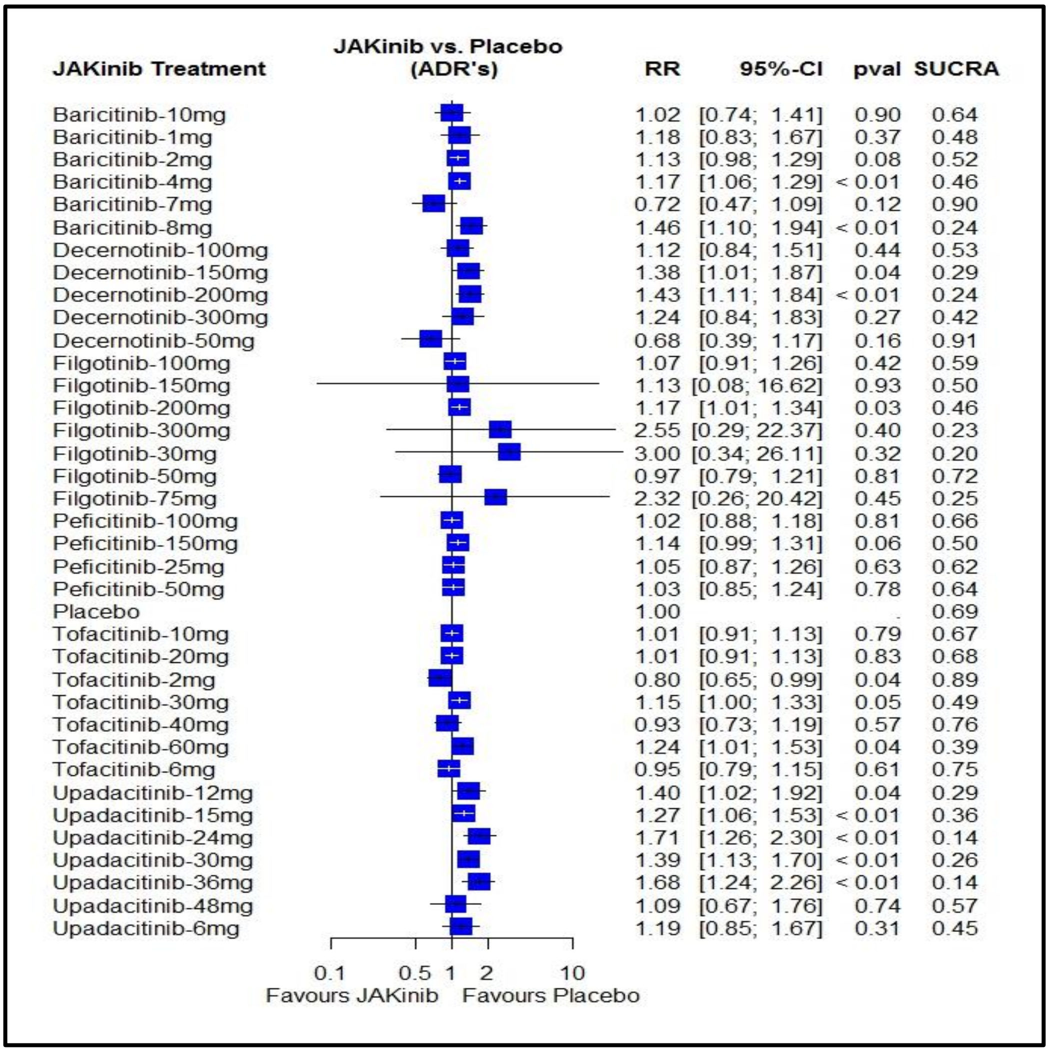

Supplementary Figure 4: Forest plot for ADR's response by different doses (mg) of JAKinibs treatment.

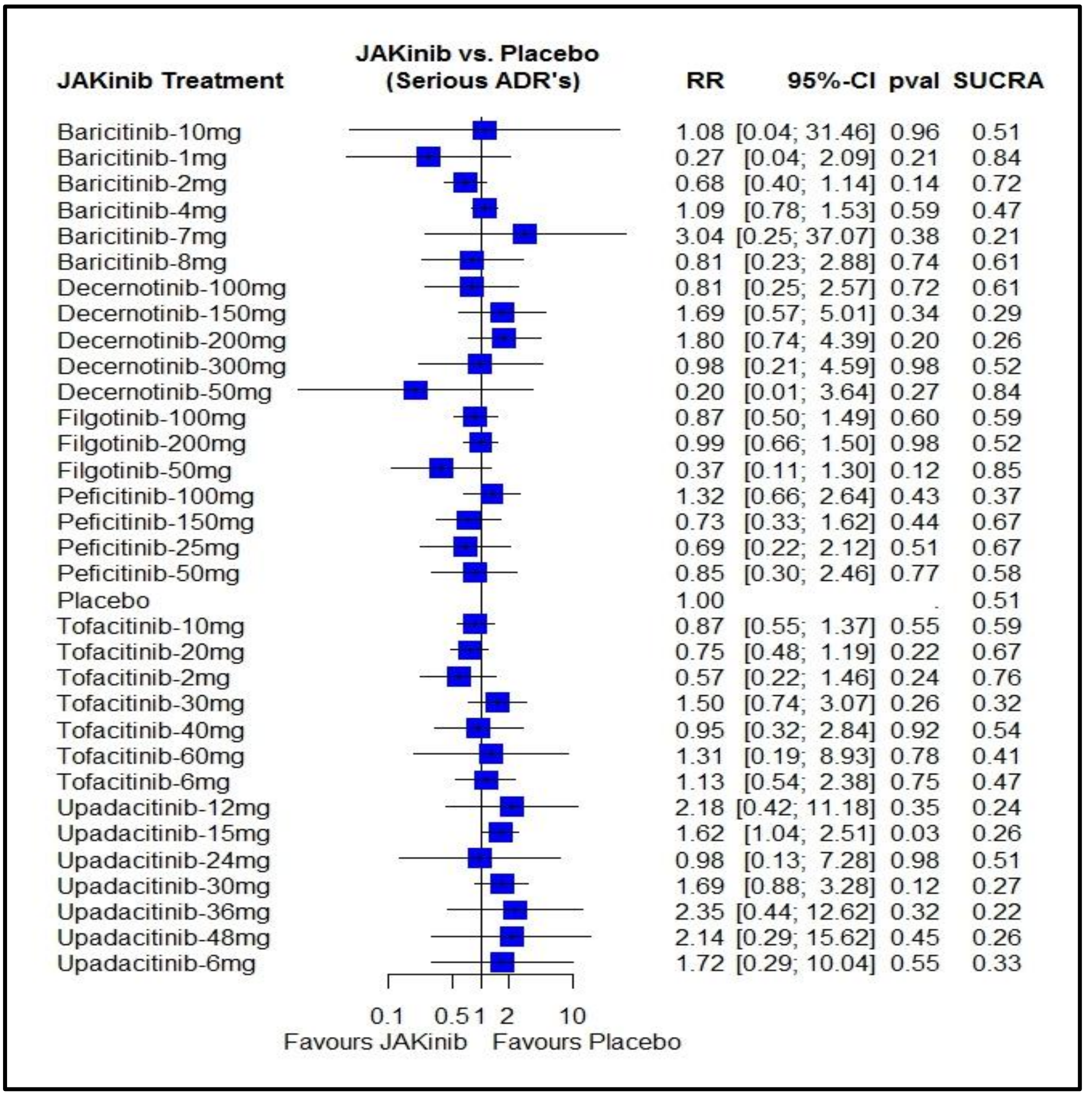

Supplementary Figure 5: Forest plot for serious ADR's response by different doses (mg) of JAKinibs treatment.

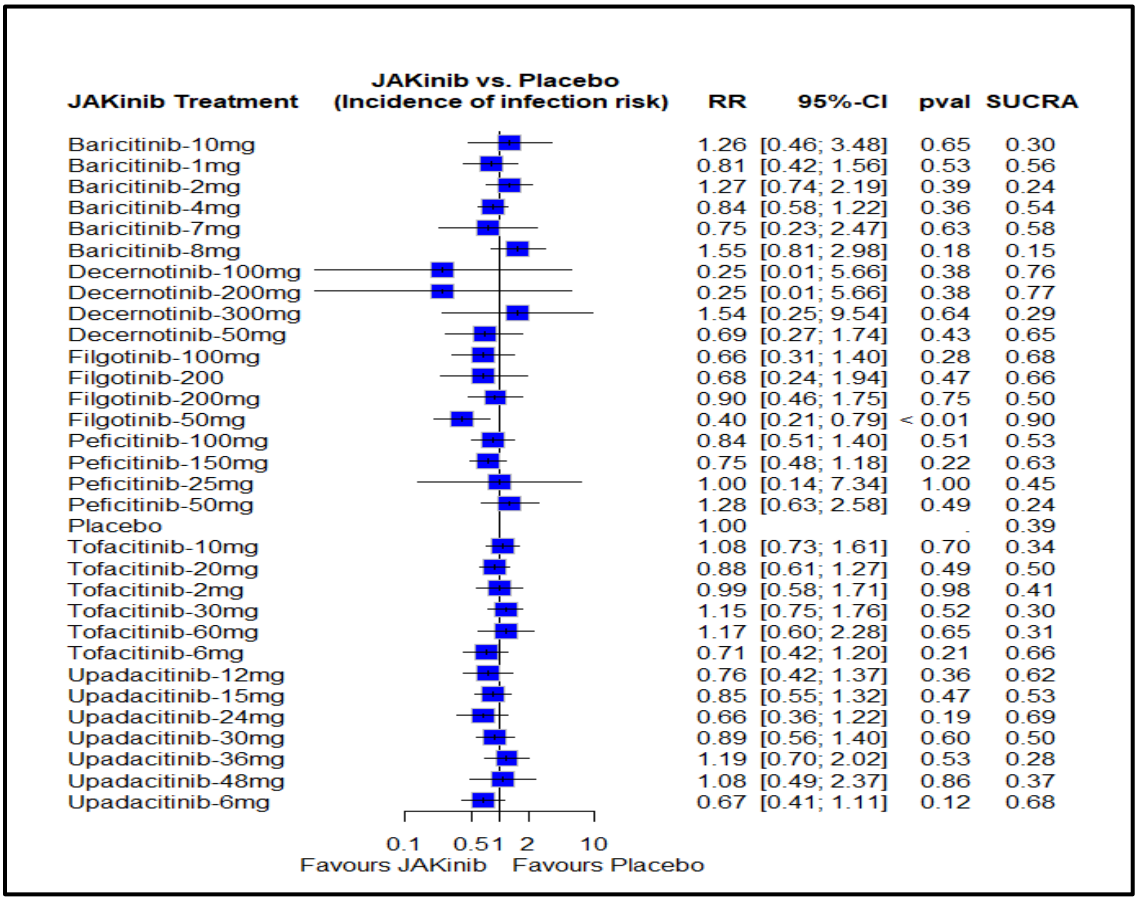

Supplementary Figure 6: Forest plot for infection risk by different doses (mg) of JAKinibs treatment.

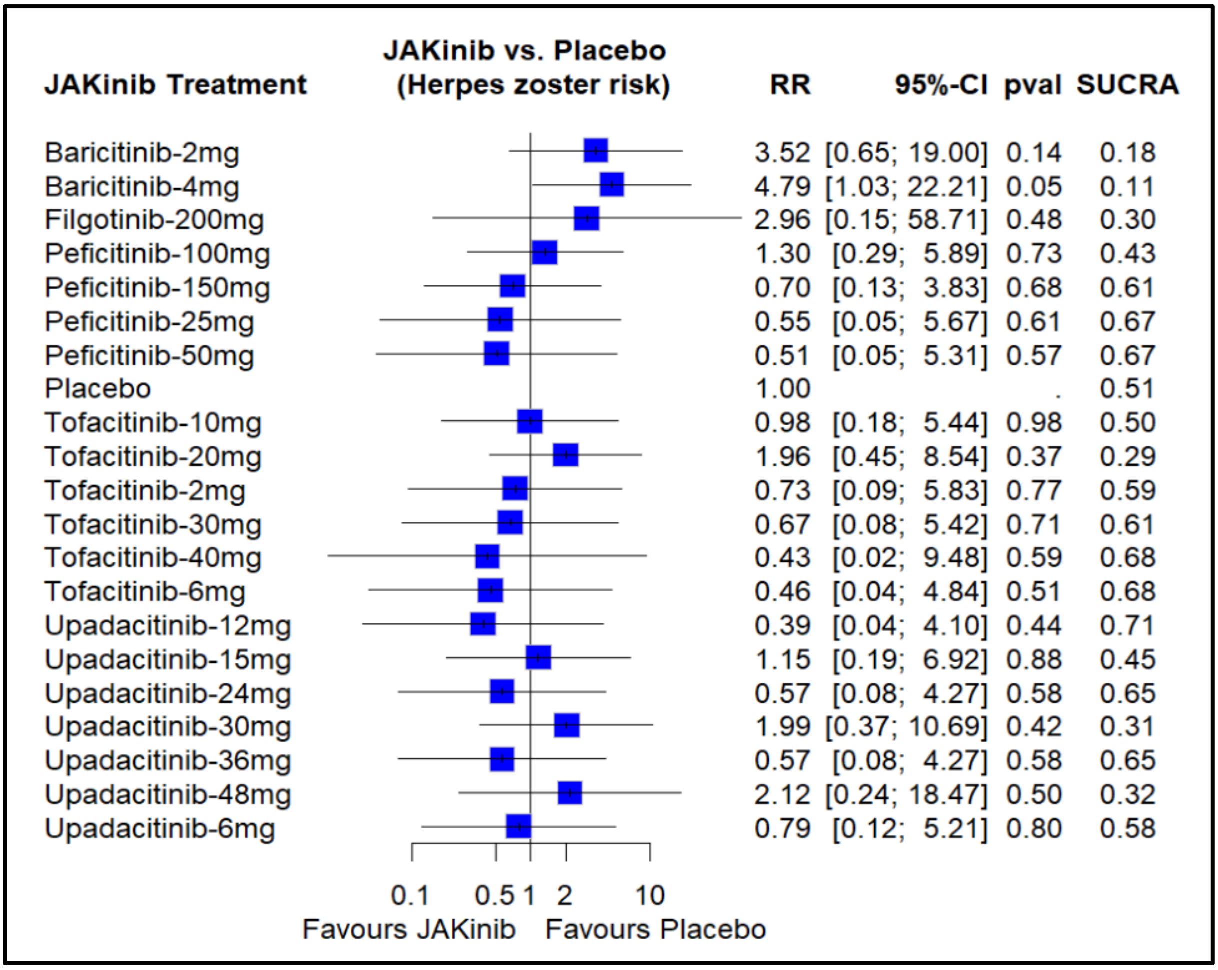

Supplementary Figure 7: Forest plot for herpes zoster risk by different doses (mg) of JAKinibs treatment.

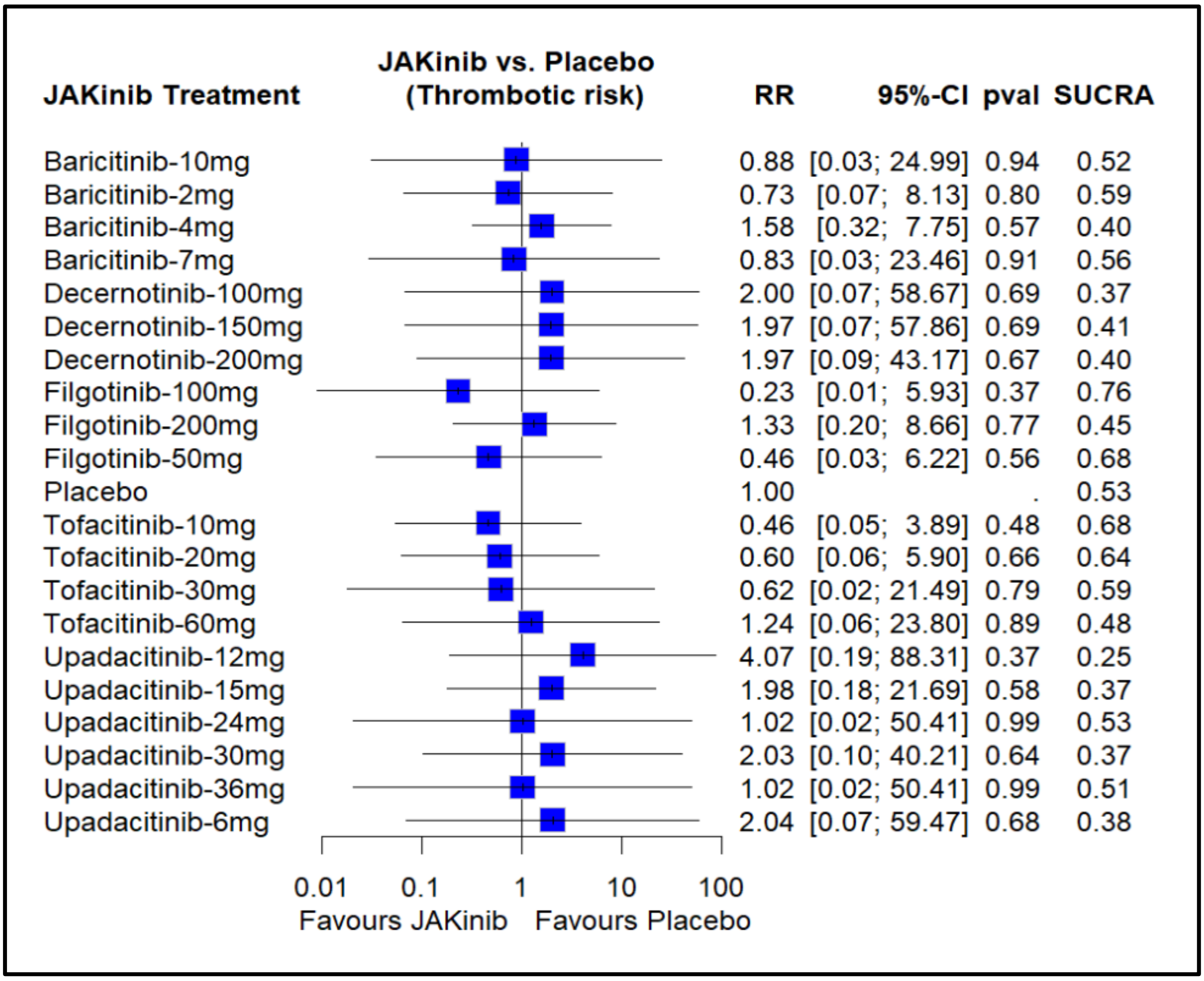

Supplementary Figure 8: Forest plot for thrombotic risk by different doses (mg) of JAKinibs treatment.

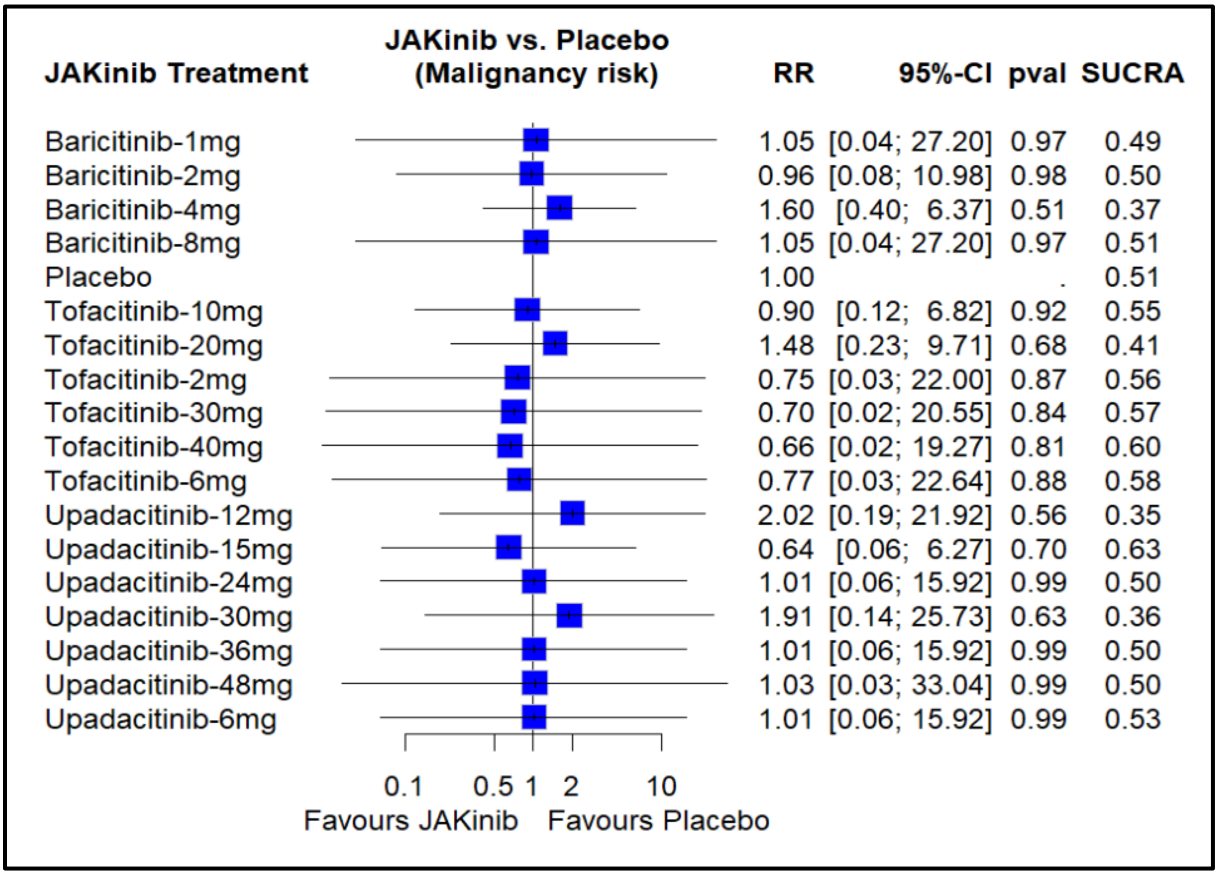

Supplementary Figure 9: Forest plot for malignancy risk by different doses (mg) of JAKinibs treatment.

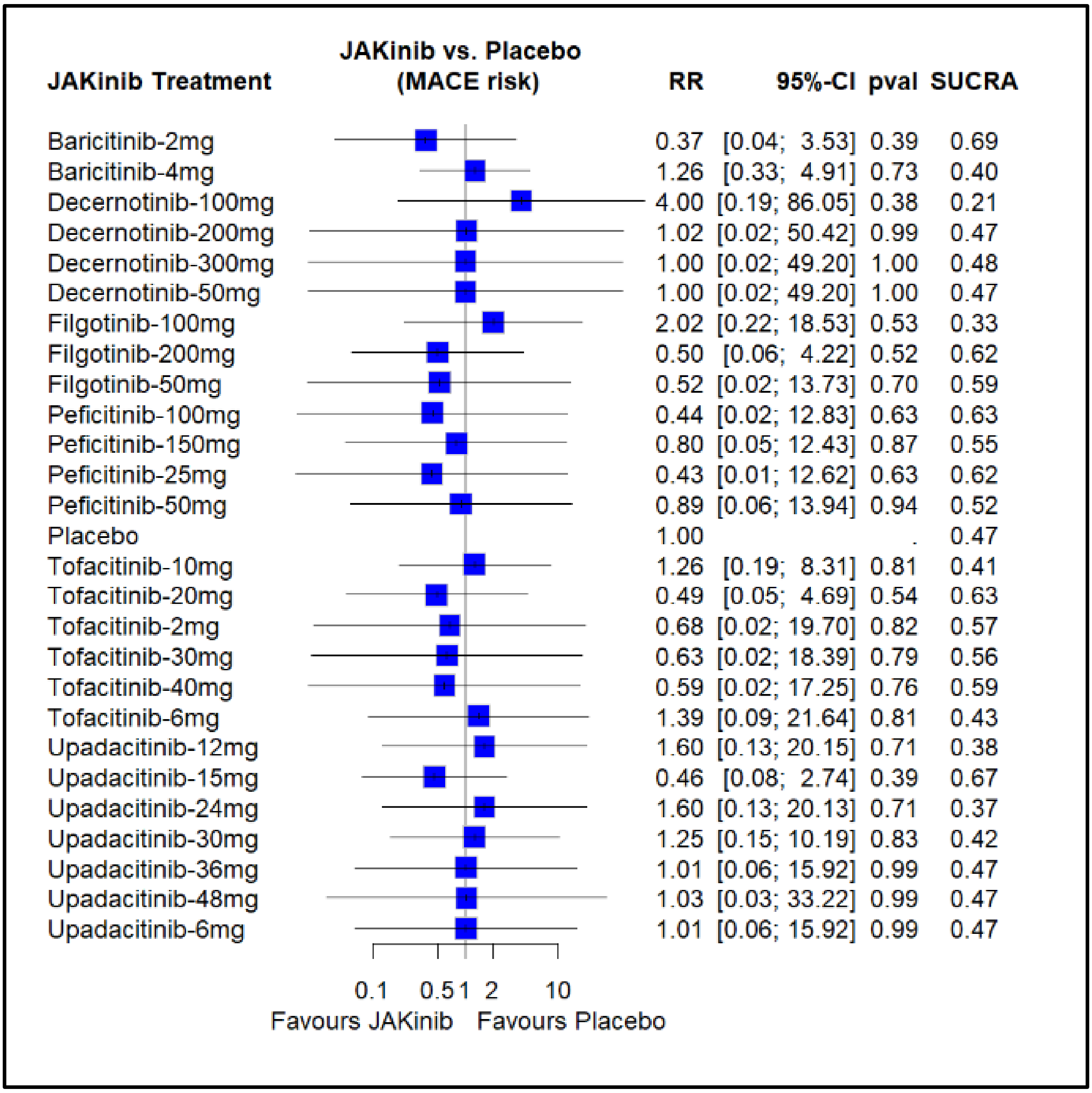

Supplementary Figure 10: Forest plot for MACE risk by different doses (mg) of JAKinibs treatment.

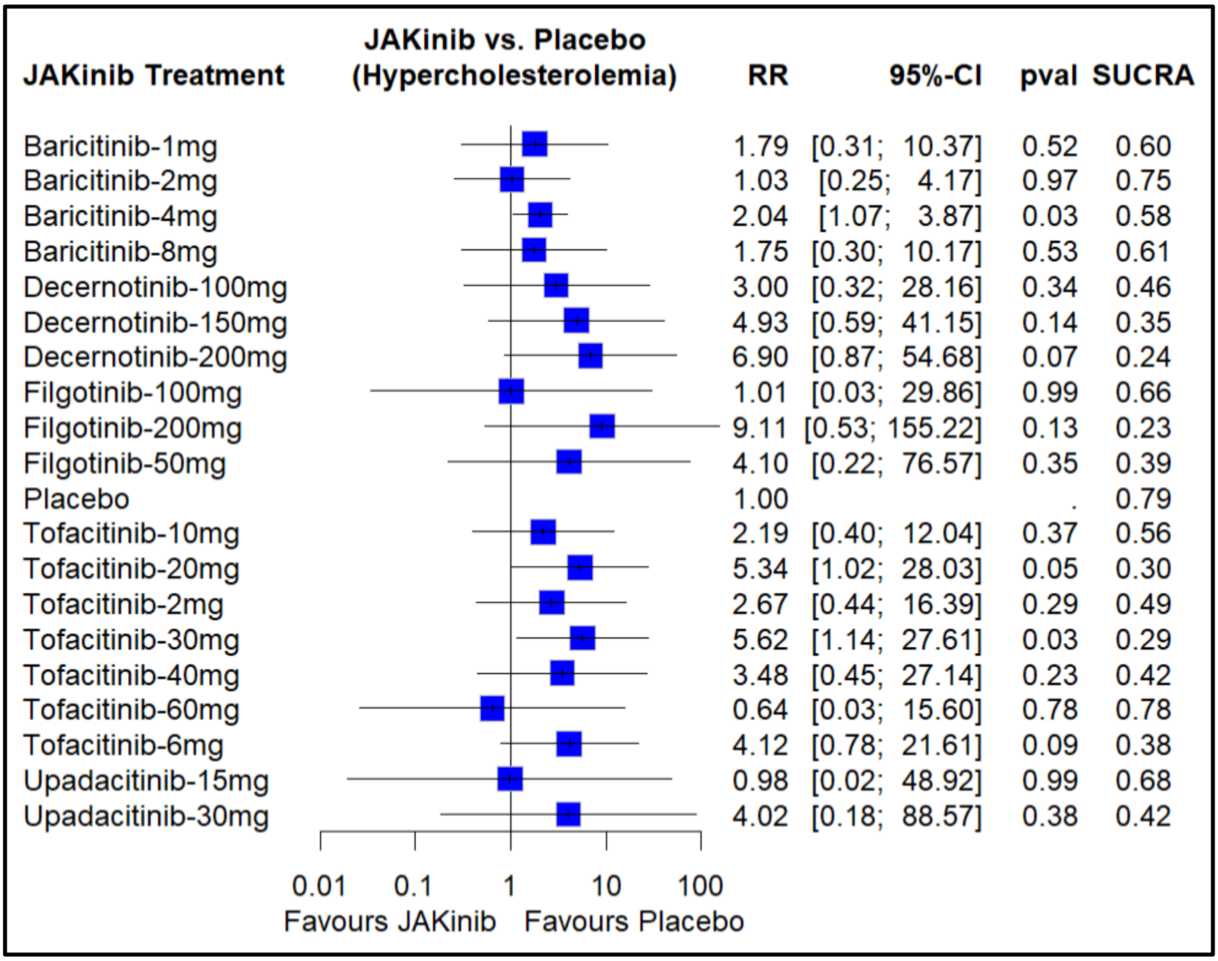

Supplementary Figure 11: Forest plot for hypercholesterolemia risk by different doses (mg) of JAKinibs treatment.

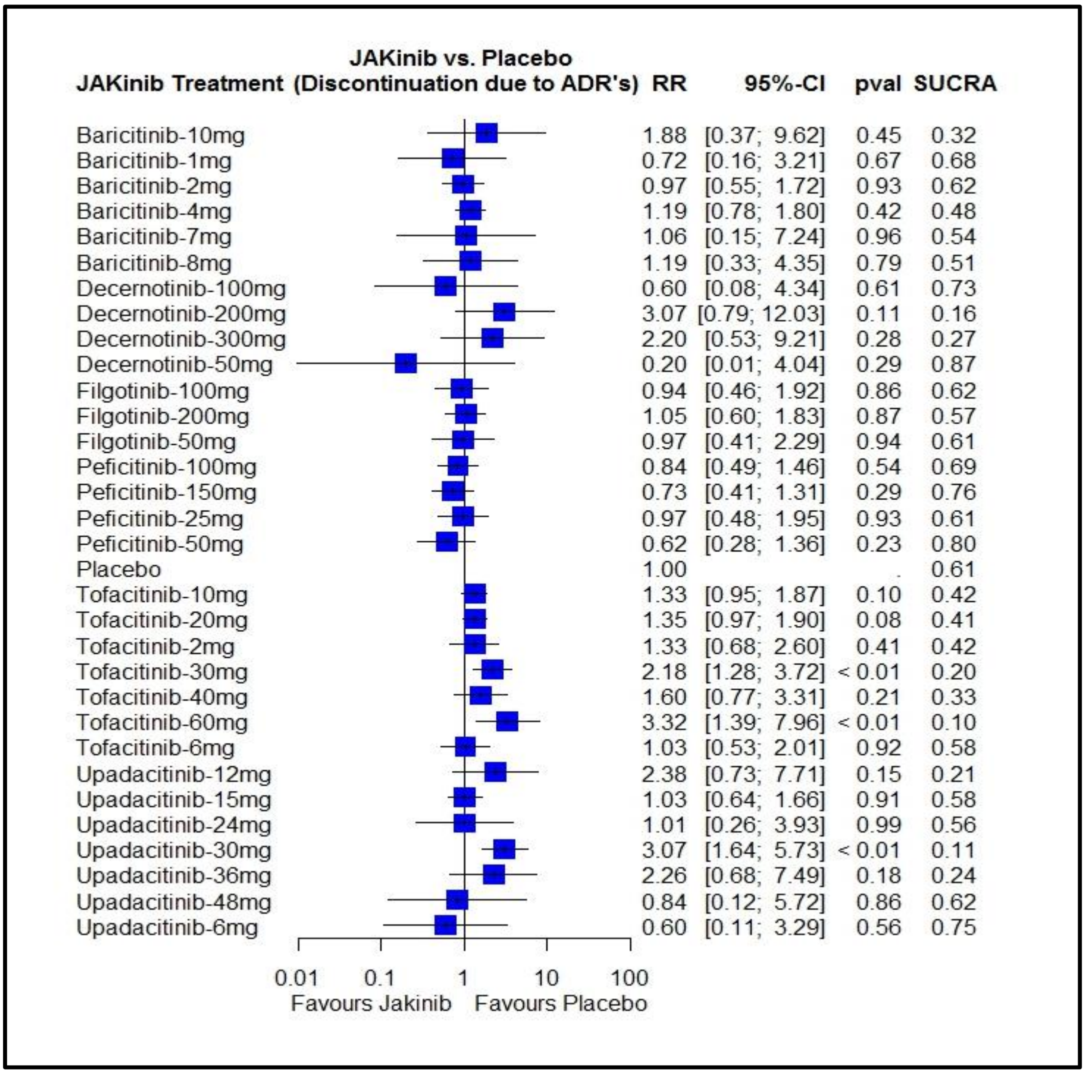

Supplementary Figure 12: Forest plot for discontinuation due to ADR's by different doses (mg) of JAKinib's treatment.

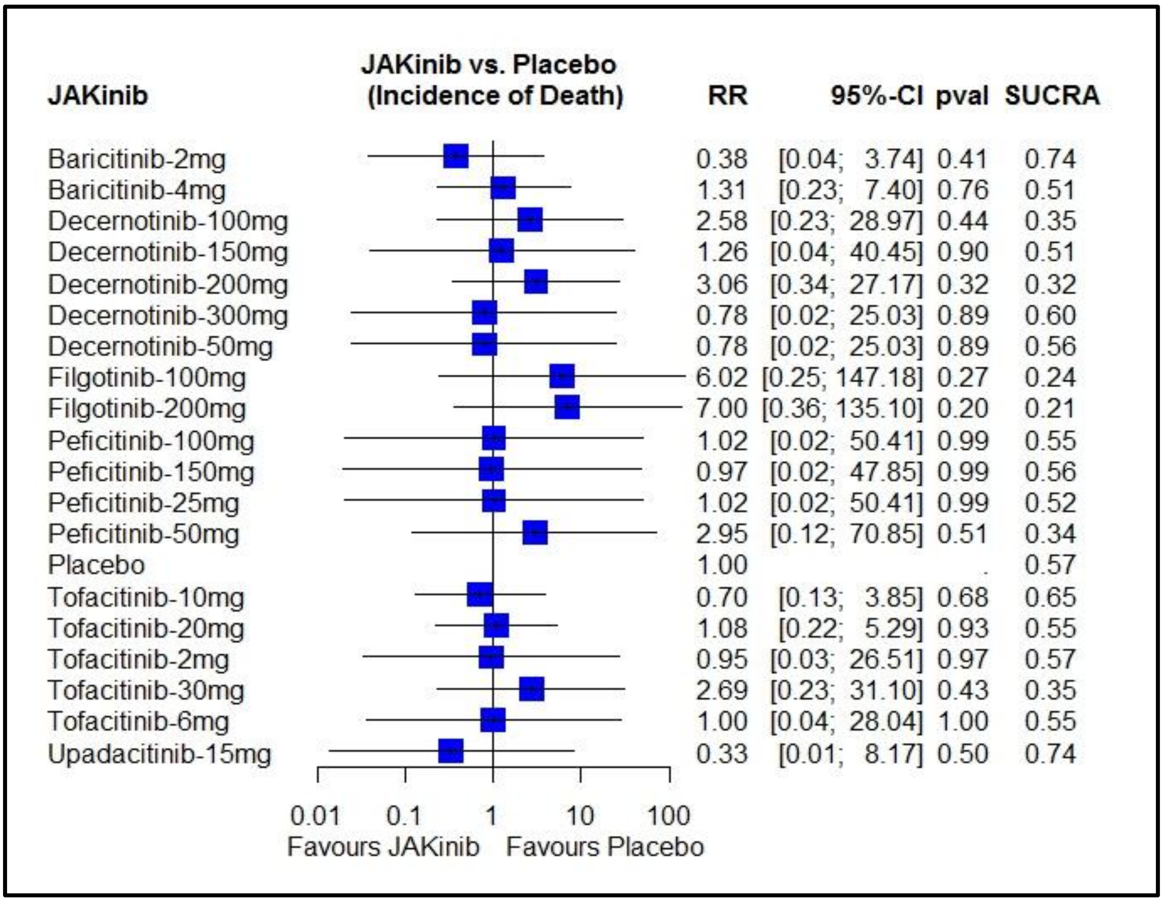

Supplementary Figure 13. Forest plot for incidence of death by by different doses (mg) of JAKinib’s treatment

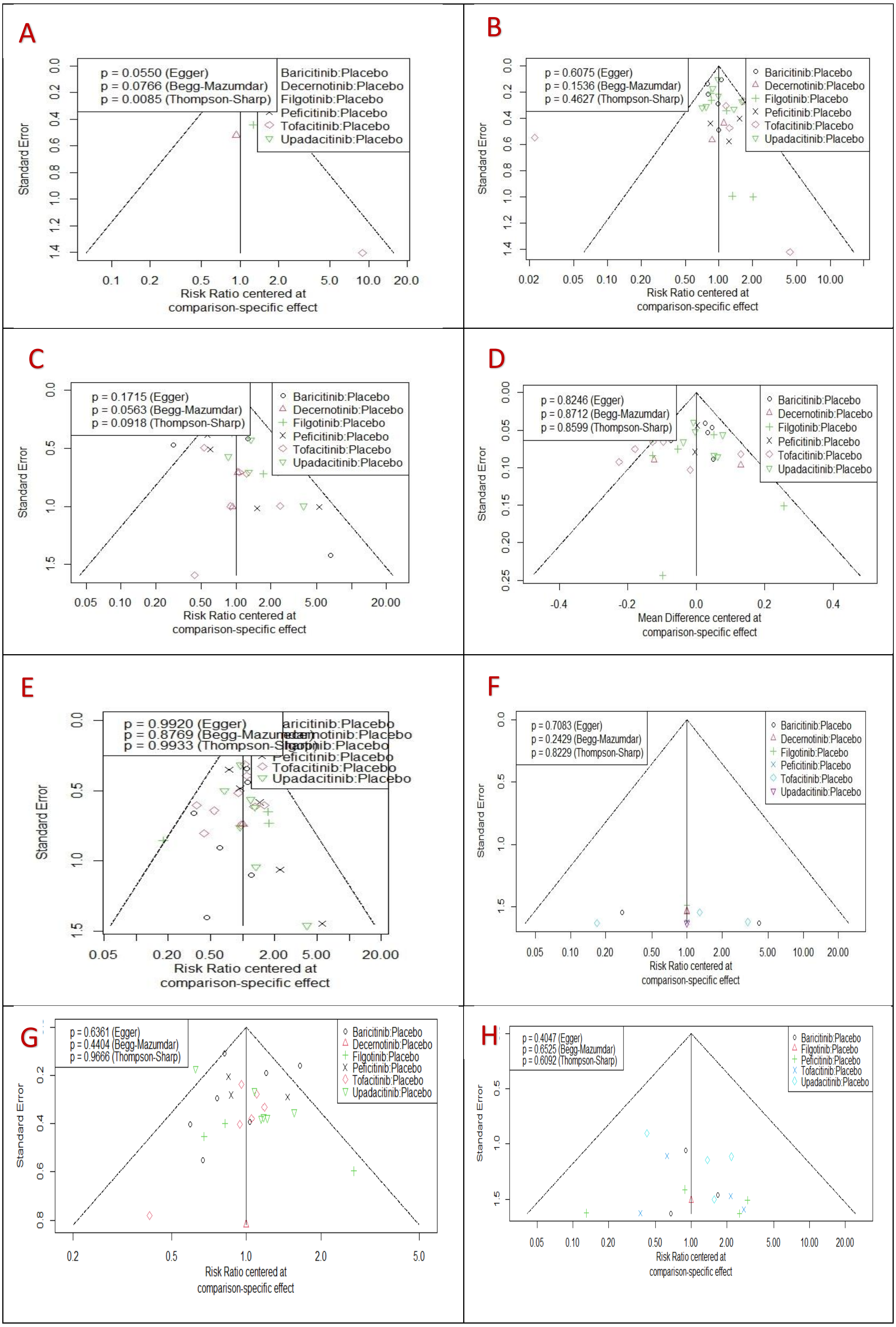

A
p = 0.0550 (Egger)
p = 0.0766 (Begg-Mazumdar)
p = 0.0085 (Thompson-Sharp)
Baricitinib:Placebo
Decernotinib:Placebo
Filgotinib:Placebo
Peficitinib:Placebo
Tofacitinib:Placebo
Upadacitinib:Placebo
Standard Error
Risk Ratio centered at comparison-specific effect
B
p = 0.6075 (Egger)
p = 0.1536 (Begg-Mazumdar)
p = 0.4627 (Thompson-Sharp)
Baricitinib:Placebo
Decernotinib:Placebo
Filgotinib:Placebo
Peficitinib:Placebo
Tofacitinib:Placebo
Upadacitinib:Placebo
Standard Error
Risk Ratio centered at comparison-specific effect
C
p = 0.1715 (Egger)
p = 0.0563 (Begg-Mazumdar)
p = 0.0918 (Thompson-Sharp)
Baricitinib:Placebo
Decernotinib:Placebo
Filgotinib:Placebo
Peficitinib:Placebo
Tofacitinib:Placebo
Upadacitinib:Placebo
Standard Error
Risk Ratio centered at comparison-specific effect
D
p = 0.8246 (Egger)
p = 0.8712 (Begg-Mazumdar)
p = 0.8599 (Thompson-Sharp)
Baricitinib:Placebo
Decernotinib:Placebo
Filgotinib:Placebo
Peficitinib:Placebo
Tofacitinib:Placebo
Upadacitinib:Placebo
Standard Error
Mean Difference centered at comparison-specific effect
E
p = 0.9920 (Egger)
p = 0.8769 (Begg-Mazumdar)
p = 0.9933 (Thompson-Sharp)
Peficitinib:Placebo
Tofacitinib:Placebo
Upadacitinib:Placebo
Standard Error
Risk Ratio centered at comparison-specific effect
F
p = 0.7083 (Egger)
p = 0.2429 (Begg-Mazumdar)
p = 0.8229 (Thompson-Sharp)
Baricitinib:Placebo
Decernotinib:Placebo
Filgotinib:Placebo
Peficitinib:Placebo
Tofacitinib:Placebo
Upadacitinib:Placebo
Standard Error
Risk Ratio centered at comparison-specific effect
G
p = 0.6361 (Egger)
p = 0.4404 (Begg-Mazumdar)
p = 0.9666 (Thompson-Sharp)
Baricitinib:Placebo
Decernotinib:Placebo
Filgotinib:Placebo
Peficitinib:Placebo
Tofacitinib:Placebo
Upadacitinib:Placebo
Standard Error
Risk Ratio centered at comparison-specific effect
H
p = 0.4047 (Egger)
p = 0.6525 (Begg-Mazumdar)
p = 0.6092 (Thompson-Sharp)
Baricitinib:Placebo
Filgotinib:Placebo
Peficitinib:Placebo
Tofacitinib:Placebo
Upadacitinib:Placebo
Standard Error
Risk Ratio centered at comparison-specific effect

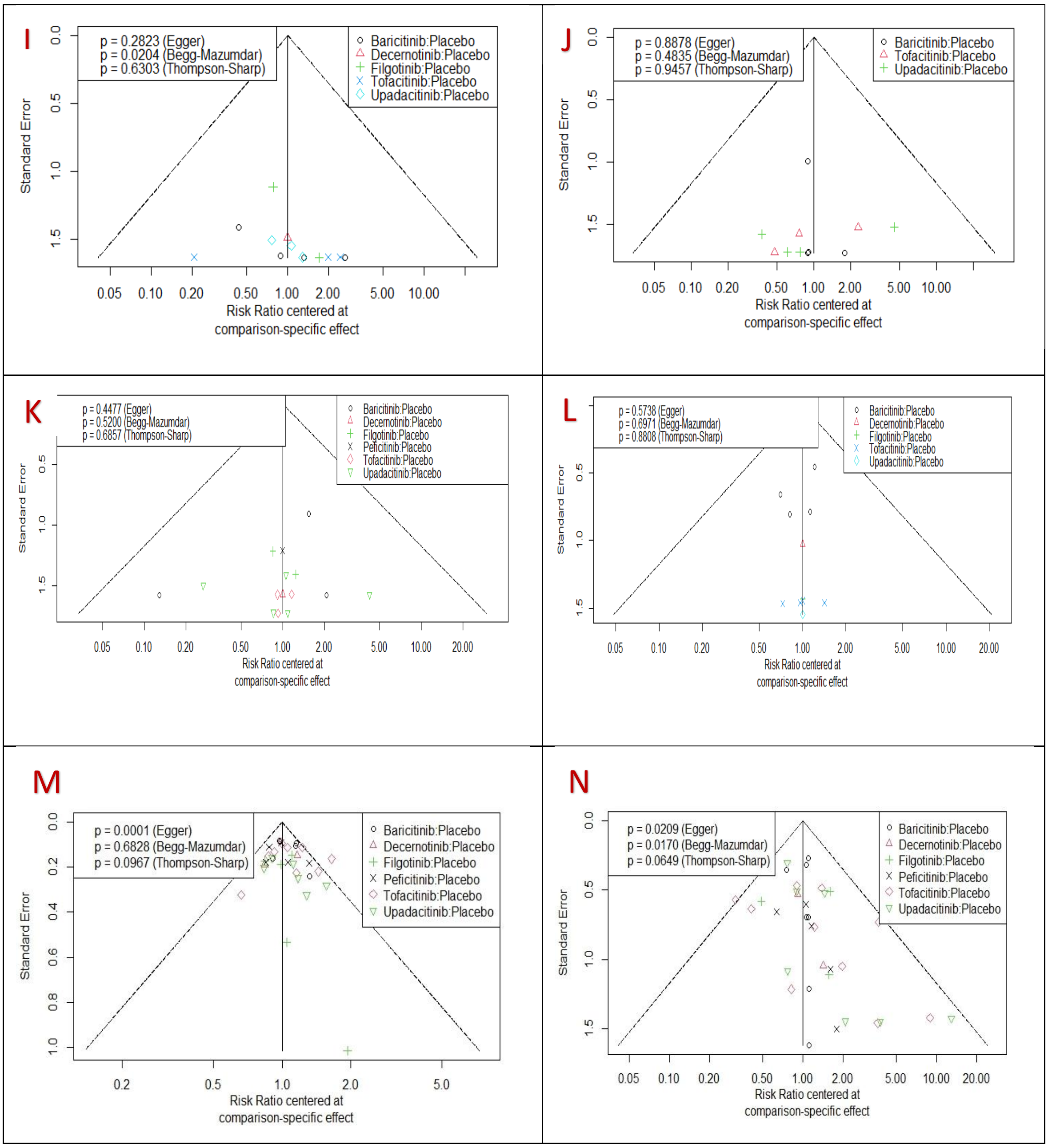

Supplementary Figure 14: (A) Funnel of ACR20 response; (B) Funnel plot of ACR50 response; (C) Funnel plot of ACR 70; (D) Funnel plot of HAQ-DI score; (E) Funnel plot of discontinuous due rate; (F) Funnel plot of incidence of death; (G): Funnel plot of infection risk; (H) Funnel plot of herpes zoster risk; (I) Funnel plot of thrombotic risk; (J) Funnel plot of malignancy risk; (K) Funnel plot of MACE risk; (L) Funnel plot of hypercholesterolemia risk. (M) Funnel plot of Incidence of ADRs; (N) Funnel plot of Serious ADRs;
